# Sex differences in temperature-related all-cause mortality in the Netherlands

**DOI:** 10.1007/s00420-021-01721-y

**Published:** 2021-06-05

**Authors:** Mireille A. Folkerts, Peter Bröde, W. J. Wouter Botzen, Mike L. Martinius, Nicola Gerrett, Carel N. Harmsen, Hein A. M. Daanen

**Affiliations:** 1 Department of Human Movement Sciences, Faculty of Behaviour and Movement Sciences, Vrije Universiteit Amsterdam, Amsterdam Movement Sciences, Van der Boechorststraat 7-9, 1081 BT Amsterdam, The Netherlands; 2 Leibniz Research Centre for Working Environment and Human Factors (IfADo), Dortmund, Germany; 3 Institute for Environmental Studies (IVM), Vrije Universiteit Amsterdam, De Boelelaan 1087, 1081 HV Amsterdam, The Netherlands; 4 Statistics Netherlands, Voorburg, The Netherlands

**Keywords:** Mortality, Temperature, Climate change, Sex differences

## Abstract

**Purpose:**

Over the last few decades, a global increase in both cold and heat extremes has been observed with significant impacts on human mortality. Although it is well-identified that older individuals (> 65 years) are most prone to temperature-related mortality, there is no consensus on the effect of sex. The current study investigated if sex differences in temperature-related mortality exist in the Netherlands.

**Methods:**

Twenty-three-year ambient temperature data of the Netherlands were combined with daily mortality data which were subdivided into sex and three age classes (< 65 years, 65–80 years,  ≥ 80 years). Distributed lag non-linear models were used to analyze the effect of ambient temperature on mortality and determine sex differences in mortality attributable to the cold and heat, which is defined as mean daily temperatures below and above the Minimum Mortality Temperature, respectively.

**Results:**

Attributable fractions in the heat were higher in females, especially in the oldest group under extreme heat (≥ 97.5th percentile), whilst no sex differences were found in the cold. Cold- and heat-related mortality was most prominent in the oldest age group (≥ 80 years) and to a smaller extent in the age group between 65–80 years. In the age group < 65 years temperature-related mortality was only significant for males in the heat.

**Conclusion:**

Mortality in the Netherlands represents the typical V- or hockey-stick shaped curve with a higher daily mortality in the cold and heat than at milder temperatures in both males and females, especially in the age group ≥ 80 years. Heat-related mortality was higher in females than in males, especially in the oldest age group (≥ 80 years) under extreme heat, whilst in the cold no sex differences were found. The underlying cause may be of physiological or behavioral nature, but more research is necessary.

## Introduction

A global increase in heat waves and cold spells have been observed over the last few decades and this increase is expected to continue due to climate change (Alexander et al. 2006; Meehl and Tebaldi 2004; Rahmstorf and Coumou 2011). Although mild cold is reportedly the dominating cause of temperature-related mortality worldwide (Gasparrini et al. 2015), thermal extremes have a profound effect on morbidity and mortality, mostly due to cardiovascular or respiratory failure (Analitis et al. 2008; Baccini et al. 2008; Costello et al. 2009). To inform policies that aim to reduce temperature-related mortality, it is important to know which subgroups of the population are especially vulnerable to this cause of death. For example, it is well known that the elderly population are most at risk for temperature-related morbidity and mortality, as their thermoregulatory function is impaired, they are often less physically fit and have more chronic illnesses and diseases (Koppe et al. 2004; Worfolk 2000).

Besides an age-related increase in risk for temperature-related morbidity and mortality, there might also be sex-related differences. Males and females differ from each other in their physiology, anthropometric characteristics, body composition and social behavior, which impact their thermoregulation (Gagnon and Kenny 2012; Kaciuba-Uscilko and Grucza 2001). Females tend to have a disadvantage in the heat due to the larger body surface to mass ratio, greater body fat percentage and lower exercise capacity (Kaciuba-Uscilko and Grucza 2001). Furthermore, in hot conditions, females have a decreased heat dissipation compared to males during intense exercise due to a lower sweating capacity (Yanovich et al. 2020). In the cold, females are at a slight advantage due to their greater body fat content which functions as insulation (Kaciuba-Uscilko and Grucza 2001; McArdle et al. 1992). A higher heat-related mortality for females and a higher cold-related mortality for males is expected due to the aforementioned physiological differences.

A number of studies investigating temperature-related mortality suggest sex is a contributing factor, with females to be more at risk during the heat than males (Åström et al. 2011; Basu 2009; van Steen et al. 2019). However, this is not fully supported in the literature (Åström et al. 2011; Basu 2009; Jiao et al. 2019; Rocklöv et al. 2014; van Steen et al. 2019). In Stockholm County (Sweden), Hong Kong (China) and Wuhan (China) a higher mortality amongst males was reported in the heat (Jiao et al. 2019; Liu et al. 2020; Rocklöv et al. 2014). In different regions in Spain, three studies reported contrasting outcomes regarding sex differences in the heat. In Galicia, a higher mortality was reported amongst females (DeCastro et al. 2011), whilst in Catalonia no sex differences were reported (Basagaña et al. 2011). In Madrid, a higher mortality amongst males in the age group between 65–75 years, and higher mortality in females in the age group older than 75 years, were reported (Díaz et al. 2002). In Hong Kong (China), Stockholm County (Sweden) and Cyprus, higher mortality in males was reported in the cold (Liu et al. 2020; Pyrgou and Santamouris 2020; Rocklöv et al. 2014), while in South East England a higher mortality in females was reported (Donaldson et al. 2019). In seven USA cities (Denver, Detroit, Minneapolis, New Haven, Pittsburgh and Chicago, and Seattle) no difference between males and females was found in the cold (O'Neill et al. 2003). Differences in temperature-related mortality between studies executed in different cities and countries may be partly related to geographical location and differences in the building environment. A previous study showed that the Minimum Mortality Temperature (MMT) is depended on geographical location with southern European cities having a higher MMT than northern European cities (Krummenauer et al. 2019). Urban areas are hotter than rural areas and in cities with tall and high density buildings with a lack of green spaces, temperatures can increase significantly more than in cities surrounded with rural areas and low buildings (Koppe et al. 2004). Furthermore, structure and insulation of buildings differ between countries, which can influence the effect outdoor temperature has on the indoor temperature where people spend most of their time (Klepeis et al. 2001). However, this does not explain the lack of consensus in the literature on sex differences in temperature-related mortality, since the distribution of males and females within a city is similar. Furthermore, less information is available about the sex differences in mortality in the cold than in the heat. Therefore, the aim of the current study was to add information to the limited and sometimes contradictory pool of data on sex differences in temperature-related mortality by analyzing a large dataset based on 23 years (1995 till 2017) of daily temperature and mortality in the Netherlands.

## Methods

### Data sources

Daily mortality in the Netherlands between 1 January 1995 and 31 December 2017 was obtained from Statistics Netherlands. The data contain the daily all-cause mortality for the total Dutch population and is subdivided into sex and three age classes: < 65 years, 65–80 years, ≥ 80 years. Daily population size for the different sexes and age classes were also included in the dataset. Temperature data were obtained from the Royal Netherlands Meteorological Institute. Hourly ambient temperature was derived from the five main weather stations throughout the Netherlands (De Bilt; Rotterdam; Schiphol Airport; Eelde and Maastricht) and averaged over time and space into average daily temperature. For the Netherlands this approach seemed acceptable as the Netherlands is rather small, 300 km from north to south and 200 km from west to east. Maximum distance from a household to the closest weather station included in the study is 100 km. Correlations of the measured temperatures between the included weather stations is very high: namely *r* > 0.967. Other studies performed in the Netherlands investigating mortality and temperature used the same approach with averaged daily temperature (Botzen et al. 2020; Ekamper et al. 2009; Huynen and Martens 2015), which gives confidence in our approach for the Netherlands.

### Statistical analysis

Data were analyzed in the statistical software R version 3.6.1. Time series of daily death counts for males and females divided into the three age classes were analyzed separately by quasi-Poisson regression allowing for over dispersion (Wood 2006). Distributed lag non-linear models (DLNM) were fitted using the dlnm package (Gasparrini 2011) applying natural cubic spline functions with eight degrees-of-freedom per year for adjusting for seasonality and long-term trend. To consider non-linear temperature effects, a quadratic B-spline function with four degrees-of-freedom was used with two equally distributed internal knots placed at 0.8 °C and 13.8 °C in combination with another spline function with three logarithmically equally distributed knots to model lagged temperature effects up to 20 days (Gasparrini and Leone 2014). In addition, day-of-week was included as covariate and log population size as offset, i.e. as predictor with coefficient fixed to one.

Mortality due to the cold or heat is quantified by attributable fractions, which is the excess mortality calculated from the Minimum Mortality Temperature (MMT) (Gasparrini and Leone 2014). The MMT is the mean daily temperature at which the lowest mortality occurs and quantifies the threshold between the cold and heat mortality slope (Folkerts et al. 2020). The MMT with SE was calculated by a search algorithm over the fitted response function (Tobías et al. 2017), which was applied separately for the time series defined by sex and age group. Heat is defined as ambient temperatures higher than the MMT and cold is defined as ambient temperatures lower than the MMT. A subdivision is made between mild and extreme cold and heat mortality attributable fractions, where mild cold and heat mortality attributable fractions are defined as the mortality between the MMT and 2.5th/97.5th percentile of the mean daily temperature, and extreme cold and heat mortality attributable fractions below and above the 2.5th/97.5th percentile (Gasparrini et al. 2015).

From the calculated attributable fractions with SE, the presence of temperature-related mortality was deduced in case the attributable fraction 95% confidence interval (CI) did not include zero. The significance of sex as potential modifier of cold- and heat-related mortality was assessed by inspection of the confidence intervals for males and females.

## Results

Table 1 shows the descriptive statistics of daily mortality in the Netherlands between 1995–2017. In the Netherlands, females reach an older age than males as can be seen by the population sizes in Table 1. Furthermore, the mean age of females is slightly higher than males; + 0.3, + 0.4, + 1.0 years for the age groups < 65, 65–80, ≥ 80 years, respectively. Therefore, daily mortality per 100,000 residents of the same sex and similar age provides a better overview of sex differences in temperature-related mortality.

**Table 1:** Distribution of daily mortality in total and per 100,000 residents in the Netherlands between 1995 – 2017 subdivided into sex and age group

Sex and age group	Daily mortality								Population size (N)	Mean age (years)	MMT (Mean ± SE (°C))	Change MMT–2.5th percentile (% (CI))	Change MMT–97.5th percentile (% (CI))
	2.5th percentile^a^		Mean		MMT^b^		97.5th percentile^c^ Total Per 100,000						
	Total	Per 100,000	Total	Per 100,000	Total	Per 100,000							
Male													
Total	206	2.6	186	2.3	173	2.1	187	2.3	8,045,742	37.9	15.4 ± 0.4	14.7 (11.5–18.0)	8.0 (5.9–10.2)
< 65	44	0.6	42	0.6	40	0.6	44	0.6	7,012,552	32.5	15.6 ± 7.5	2.4 (− 3.5 to 8.7)	4.8 (0.6–9.1)
65–80	81	10.2	73	8.8	68	8.2	74	9.2	837,818	70.9	16.4 ± 1.6	17.9 (11.8–24.5)	4.0 (0.5–7.5)
≥ 80	80	42.8	71	36.2	63	32.2	69	36.5	195,373	84.2	14.8 ± 2.0	19.2 (14.1–24.5)	14.6 (10.9–18.5)
Female													
Total	216	2.7	196	2.6	183	2.1	203	2.5	8,212,706	40.0	15.1 ± 0.2	18.3 (15.1–21.6)	14.3 (12.0–16.6)
< 65	30	0.4	29	0.5	28	0.4	30	0.4	6,830,253	32.8	15.7 ± 9.4	7.4 (0.0–15.4)	2.0 (− 2.8 to 7.1)
65–80	55	5.7	51	5.7	48	4.9	52	5.4	983,848	71.3	16.1 ± 3.3	16.2 (9.0–23.9)	4.4 (0.2–8.8)
≥ 80	130	33.2	116	33.4	106	26.3	120	30.8	398,605	85.2	15.0 ± 0.2	21.9 (17.8–26.2)	21.1 (18.1–24.2)

The 2.5th and 97.5th percentile of the mean daily temperature represent the cut-off for respectively extreme cold and heat. Daily mortality shown for the 2.5th and 97.5th temperature percentiles are the averaged values below and above these percentiles, respectively, and daily mortality shown for the Minimum Mortality Temperature (MMT) is the averaged value within the standard error. Population size (N), mean age (years), MMT (°C) and the change in mortality of the 2.5th and 97.5th percentile, respectively, compared to the MMT as estimated by the distributed lag non-linear model (DLNM) are shown per sex and age group

^a^ < − 1.8 °C

^b^Minimum Mortality Temperature

^c^ > 21.5 °C

**Fig. 1:**
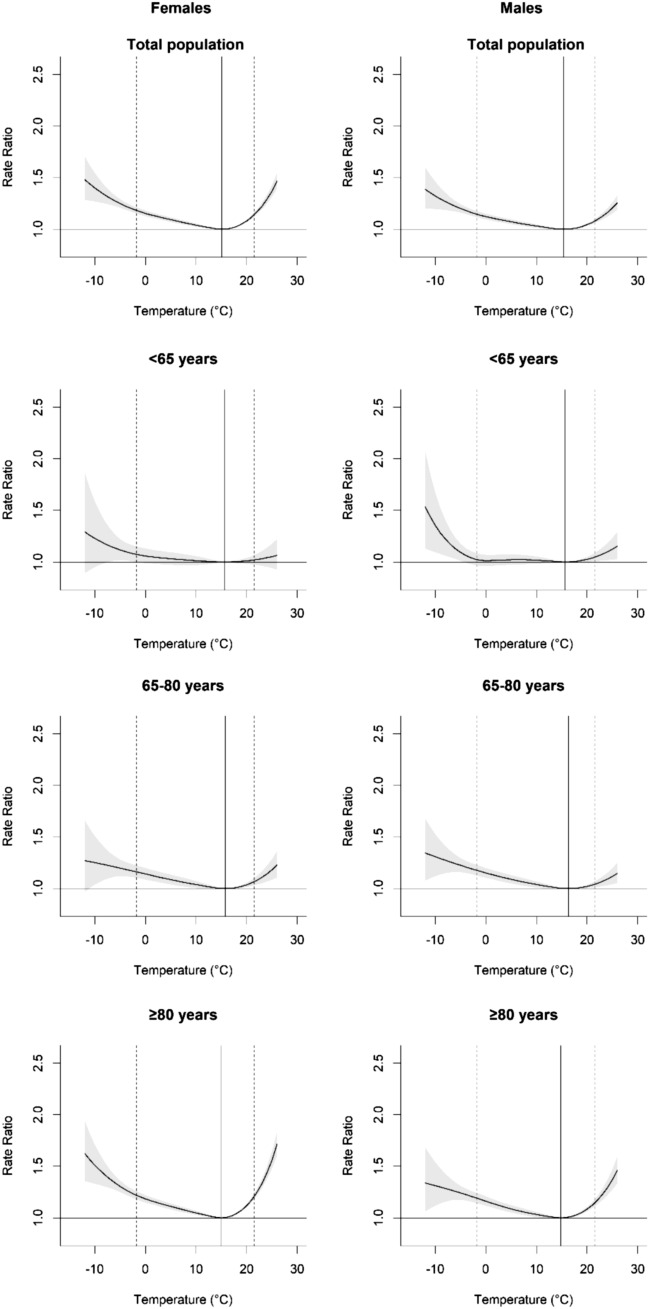
The overall cumulative exposure–response associations of daily mean temperature (°C) and daily mortality in the Netherlands between 1995–2017 subdivided into males and females for the total population and separated for three different age groups (< 65, 65–80, ≥ 80 years). The vertical solid lines represent the Minimum Mortality Temperature (MMT) and the dashed lines the 2.5th and 97.5th percentile of the mean daily temperature, respectively. Gray-shaded error bands indicate the 95% confidence interval

**Fig. 2:**
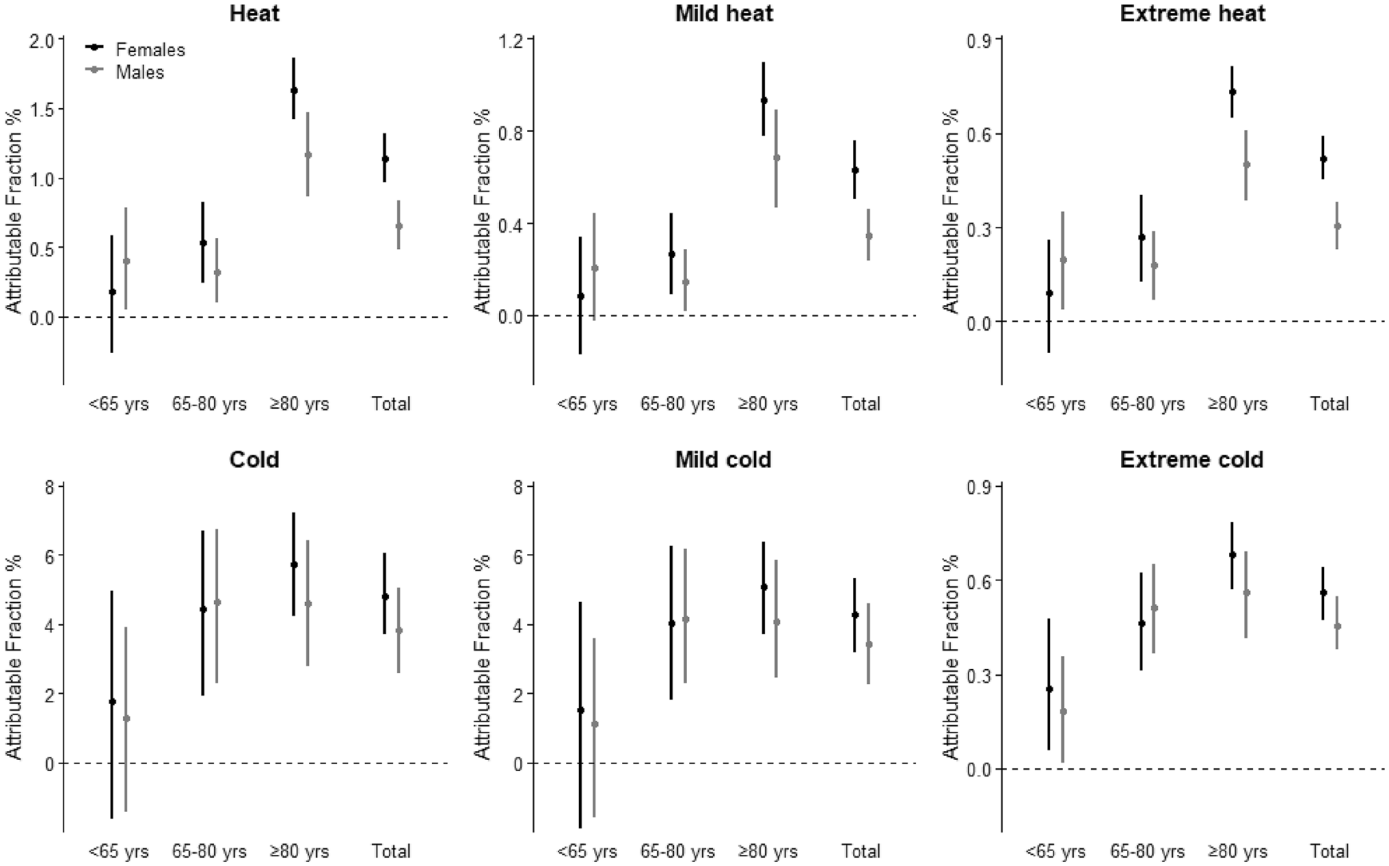
Daily mortality attributable to the heat and cold in the Netherlands between 1995–2017 subdivided into males and females for the total population and separated for three different age groups (< 65, 65–80, ≥ 80 years). Heat is defined as ambient temperatures higher than the Minimum Mortality Temperature (MMT) and cold is defined as ambient temperatures lower than the MMT. Mild cold/heat is defined as the temperature between the MMT and 2.5th/97.5th percentile of the mean daily temperature, and extreme cold/heat as the temperature below and above the 2.5^th^/97.5^th^ percentile. Vertical error bars indicate the 95% confidence interval. Please note that the y-axes are not identically scaled

Mortality due to the cold was greater than to the heat in both males and females. In the cold there was an increase in mortality from the MMT to the 2.5th percentile for males of 14.7% and for females of 18.3%. In the heat the increase in mortality from the MMT to the 97.5th percentile was 8.0% for males and 14.3% for females. The highest increase in mortality was in the oldest age group (≥ 80 years) with an increase for males and females of 19.2% and 21.9% respectively in the cold, and of 14.6% and 21.2% respectively in the heat. Especially in the heat there was a large difference between the oldest age group (≥ 80 years) and the age groups < 65 and 65–80 years. This is also shown in Figs. 1 and 2. Figure 1 shows the overall exposure–response associations of daily mean temperature (°C) and daily mortality cumulating all lagged effects in the Netherlands between 1995–2017. Figure 2 shows the daily mortality attributable fractions in the heat and cold in the Netherlands between 1995 and 2017. Data are subdivided into males and females for the total population and separated for three different age groups (< 65, 65–80, ≥ 80 years). In the age group < 65 years temperature-related mortality was non-significant; only for males in the heat, there appears to be a small temperature effect on mortality, which was comparable in size to that for males aged 65–80 years.

Overall mortality per 100,000 residents was higher in males in the heat for the age groups 65–80 years and ≥ 80 years (Table 1), but the increase in mortality from the MMT was higher for females with 4.0% and 4.4% in the age group 65–80 years and 14.6% and 21.1% in the age group ≥ 80 years for males and females respectively. Inspecting the confidence intervals for males and females in Fig. 2 indicates that in mild and extreme heat for the total population the differences between males and females were significant, but age-specific differences were significant only for the age group ≥ 80 years in extreme heat. No significant differences occurred in cold-related mortality between males and females.

## Discussion

Mortality in the Netherlands represents the typical V- or hockey-stick shaped curve with a higher daily mortality in the cold and heat than at milder temperatures in both males and females, especially in the age group ≥ 80 years. There were no sex differences in cold-related mortality in this study, but females appear to be more susceptible than males to both mild and extreme heat when considering all age groups combined. This is still the case after correcting for population size, which is important as females tend to live longer than males, which results in twice as many females than males in the Netherlands in the age group ≥ 80 years. Age-specific significant sex differences were only observed for the oldest group in extreme heat, but not in mild heat as for the total population, probably due to lower statistical power associated with fewer death events in the age-specific time series (Armstrong et al. 2020). These findings are in line with the majority of previous studies, from various cities/countries with different MMT and climates (Bettaieb et al. 2020; Ling-Shuang et al. 2020; van Steen et al. 2019; Yu et al. 2010). The MMT of different studies and countries can differ due to the analytical method used (Folkerts et al. 2020), but also because of differences in climate. The MMT decreases from southern to northern European countries and is strongly related to the mean ambient temperature (Krummenauer et al. 2019). In the south of Europe the MMT can be as high as 26.5 °C to 28 °C, while in the north of Europe it can be as low as 16.0 °C to 17.5 °C (Krummenauer et al. 2019). In the current study we even found an average MMT of 15.3 °C on average. Different climates reported in previous studies are, for example, Hunan (China), which has a humid subtropical climate, Galicia (Spain), which has a mild oceanic climate and Belgrade (Serbia), which has a continental climate (Bogdanović et al. 2013; Central Intelligence Agency–The World Factbook 2021; DeCastro et al. 2011; Ling-Shuang et al. 2020). To our knowledge, this is the first study to explore sex differences in temperature-related mortality in a temperate marine climate, as observed in the Netherlands. Our results contribute to the existing body of literature and provide evidence that, alongside other climates, females appear to be more vulnerable to heat-related mortality.

The increasing risk for heat-related mortality as people age, may partly be explained by the age-related physiological changes. McGinn et al. (2017) showed that whole-body heat loss decreases with 4% each decade after the age of 20 years in healthy adults. The reduction in the ability to lose heat with aging is for the largest part explained by the lower overall sweat rate in older adults, as evaporative heat loss is the strongest avenue of heat loss from the human body (Balmain et al. 2018; McArdle et al. 2014). To the authors’ knowledge, no studies have investigated the age-related physiological decline between males and females in their ability to lose heat. However, it is known that females sweat less than males, and older females sweat less than younger females, indicating that the ability of older females to lose heat from the body is the lowest (Daanen and Herweijer 2015; Yanovich et al. 2020). Heat puts considerable stress on the cardiovascular system, especially in older individuals who rely on a greater percentage of their maximum heart rate to increase cardiac output during whole-body heat stress than young adults, indicating greater cardiovascular strain (Kenney et al. 2014). Not surprisingly, one of the main causes of heat-related mortality is related to a cardiovascular disorder (Åström et al. 2011). Cardiovascular strain is reportedly higher in females, potentially explaining their higher mortality risk in the heat (Achebak et al. 2019; Yanovich et al. 2020).

Furthermore, the behavior of older males and females may also influence the higher risk for mortality in the heat. It has been reported, that older adults are less willing to use adaptive cooling strategies, potentially due to reduced sensitivity to thermal comfort, lack of social support or not wanting to be seen as ‘old’ and ‘vulnerable’ (Tan et al. 2020). Some studies suggest women initiate thermal behavior to cool down earlier and use it to a greater extent than males (Corbett et al. 2020; Vargas et al. 2019). However, these studies are conducted with young subjects and it is not known if this difference still persists in older adults. Van Steen et al. (2019) stated that females often outlive their male partners and therefore live alone, which has been reported as a contributing factor as physical and social isolation is highly correlated to heat-related mortality. It was also reported that females tend to be less active in general, but more active in the household (Lee 2005). Continuing these activities during heat waves while being less physically fit puts females more at risk for overheating and cardiovascular strain than males (van Steen et al. 2019). Furthermore, it was mentioned that females often have a lower income which is associated with less quality housing and often no air conditioning; resulting in higher indoor temperatures (van Steen et al. 2019). In the Netherlands housing quality is generally of a high standard and the houses are well insulated (Planbureau voor de Leefomgeving–Balans van de Leefomgeving 2016). Higher insulation may hamper heat removal during the summer, thus increasing indoor heat stress. However, a previous study showed that insulation only plays a minor role in the risk of overheating and when ventilated proper insulation even lowers the severity and risk of overheating (Fosas et al. 2018). Furthermore, the ownership of air conditioning units is not that common in the Netherlands (The Japan Refrigeration and Air Conditioning Industry Association 2018; Planbureau voor de Leefomgeving–Balans van de Leefomgeving 2016). Therefore it seems unlikely that a lower income of females will explain the sex differences in heat-related mortality in the Netherlands when considering housing quality and use of air conditioning.

More research is needed to determine these sex differences in heat-related mortality from both a physiological and behavioral perspective. The limited number of physiological studies with female participants may be contributing to our poor understanding of their response to the heat. A better understanding of these differences will allow for better targeted heat policies for both males and females and potentially lower heat-related morbidity and mortality across the general population. For example, in 2007 a heat health warning system (HHWS) was developed in the Netherlands which is activated if there is a high chance of five consecutive days with an ambient temperature exceeding 27 °C (Casanueva et al. 2019; Lowe et al. 2011). More attention for females in the HHWS could be a next step at decreasing the negative effects of heat for females.

In the cold a significant higher effect on mortality was found for the age groups 65–80 and ≥ 80 years, but no sex differences were observed. Cold-related mortality in older adults has mostly a cardiovascular, respiratory, and cerebrovascular origin (Analitis et al. 2008). The observed absence of any sex differences in the cold is in line with a previous study done in the USA (O'Neill et al. 2003). However, other studies have reported a higher mortality in males or females in the cold (Donaldson et al. 2019; Liu et al. 2020; Pyrgou and Santamouris 2020; Rocklöv et al. 2014). Sex differences in the cold seem to be less prominent than in the heat, as there is no consensus in outcome of different studies. Potentially this is the case as the physiological disadvantage of males, e.g. the lower body fat content, can easily be compensated with thermoregulatory behaviors such as adding an extra layer of clothing (Kaciuba-Uscilko and Grucza 2001). A previous study showed that males wear 0.14 clo more than females, suggesting this may indeed be the case (Donaldson et al. 2001). Furthermore, adaptive behavior to the cold may differ between countries and therefore result in different outcomes in mortality. One specific difference in adaptive behavior to the cold between regions is the wearing of gloves, hats and scarfs, which is reported to be less frequent in the southern regions of Europe (Donaldson et al. 2001). Another explanation for the lack of consistency in sex differences in cold-related mortality between studies is the level of income of the elderly population in a country. People with higher incomes can afford better insulated houses and energy to warm their houses in winter, which reduces their exposure to the cold compared to people with lower incomes (McKee 1989). However, not much is known about these potential behavioral differences between countries and future research should focus on this, which could be used to adapt policies regarding behavior in the cold.

## Limitations

As our analyses concern all-cause mortality only, this limits our interpretation of the specific causes of cold- and heat-related mortality. Furthermore, the temperature data of the five weather stations in the Netherlands were averaged and considered to be indicative for the weather conditions in the Netherlands. Ambient temperature at the location where a person passed away can differ from the mean ambient temperature. However, this difference is considered to be minimal in a small country like the Netherlands with a mean standard deviation of 0.7 °C between weather stations. In the current study only mean ambient temperature is used to represent the thermal environment and no other parameters like humidity and solar radiation. A previous study showed no correlation between mortality and humidity, indicating that potentially humidity had limited influence in the current study as well (Armstrong et al. 2019). However, future studies should focus more in depth what the effect is of different environmental parameters, like humidity and solar radiation, on the higher mortality in females, preferably using data at a regional or local level. Moreover, no correction was made for the mean age of the males and females within each age group. However, as shown in Table 1, mean age within each age group is almost similar. Therefore, it is assumed to have no effect on the reported results.

## Conclusion

Mortality in the Netherlands represents the typical V- or hockey-stick shaped curve with a higher daily mortality in the cold and heat than at milder temperatures in both males and females, especially in the age group ≥ 80 years. Heat-related mortality in the Netherlands was higher in females than in males, especially in the oldest age group (≥ 80 years) under extreme heat, whilst in the cold no sex differences were found. The underlying cause may be of physiological or behavioral nature, but more research is necessary.

## Data Availability

The datasets generated for this study are available on request to the corresponding author.

## Code availability

The R script used for this study is available on request to the corresponding author.
